# Supplementary material for: Particulate Matter-Induced Neurotoxicity: Unveiling the Role of NOX4-Mediated ROS Production and Mitochondrial Dysfunction in Neuronal Apoptosis
Source: Int J Mol Sci. 2024 Jun 1;25(11):6116. doi: 10.3390/ijms25116116 (PMC11172693; doi:10.3390/ijms25116116)
Supplement: Supplementary file 1 [file ijms-25-06116-s001.zip › Supplementary Figures.pdf]

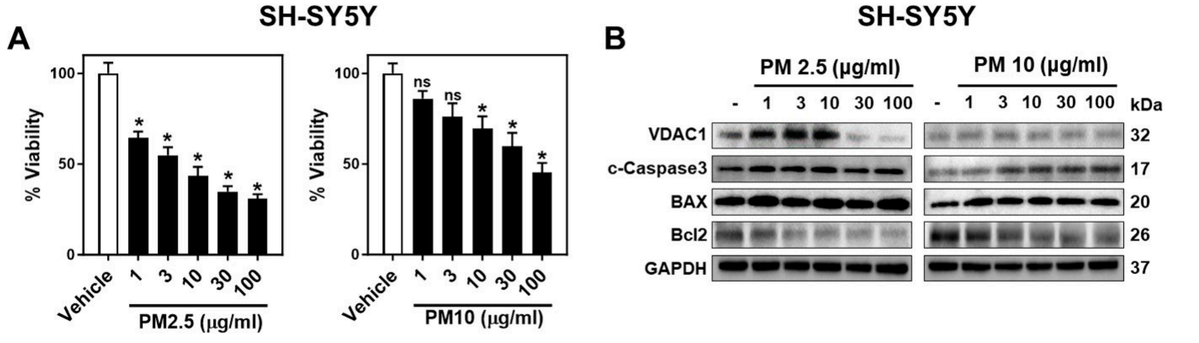

Supplementary Figure S1. PM2.5 and PM10 induce cell death through mitochondria-mediated apoptosis in SH-SY5Y. (A) Effect of PM2.5 (left) and PM10 (right) on the cell viability of SH-SY5Y cells assessed by the MTT assay. Cells were incubated with different concentrations (1, 3, 10, 30, 100 μg/ml for 24 h) and PBS was used as the control (vehicle). Data were presented as mean ± SEM, $^*p \leq 0.01$ vs. Vehicle. Data were analyzed by one-way ANOVA (B) WB showing the effects of PM2.5 (left) and PM10 (right) treatment on VDAC1, cleaved (c)-caspase3, BAX, and Bcl2 in SH-SY5Y cells at various concentrations (1, 3, 10, 30, 100 μg/ml for 24 h). GAPDH was used as a loading control.

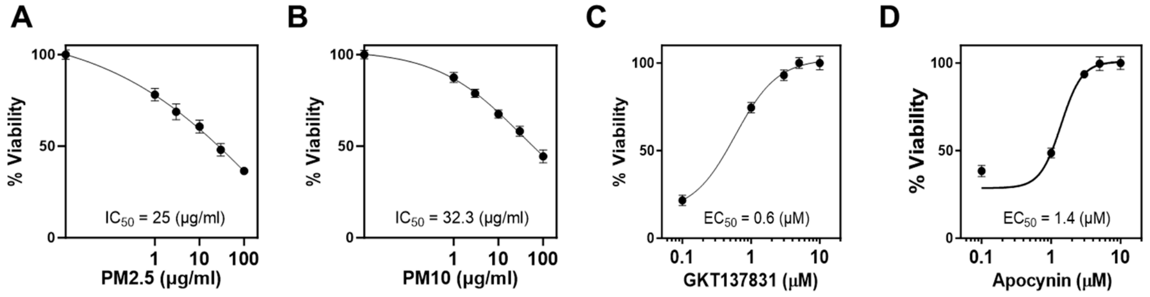

Supplementary Figure S2. Dose-dependent effects of PM2.5, PM10, GKT137831, and Apocynin on cell viability in Neuro-2A. (A-B) Nonlinear regression analysis was performed to determine the IC_{50} values, using the viability data re-analyzed from Figure 1A. The IC_{50} for (A) PM2.5 or (B) PM10 was found to be 25 μg/ml and 32.3 μg/ml, respectively. (C-D) Nonlinear regression analysis was conducted to determine the EC50 values of GKT137831 and Apocynin in the context of PM2.5 (100 μg/ml)-induced reduction in cell viability. The EC_{50} for (C) GKT137831 or (D) Apocynin was found to be 0.6 μM and 1.4 μM, respectively. Data were presented as mean ± SEM.

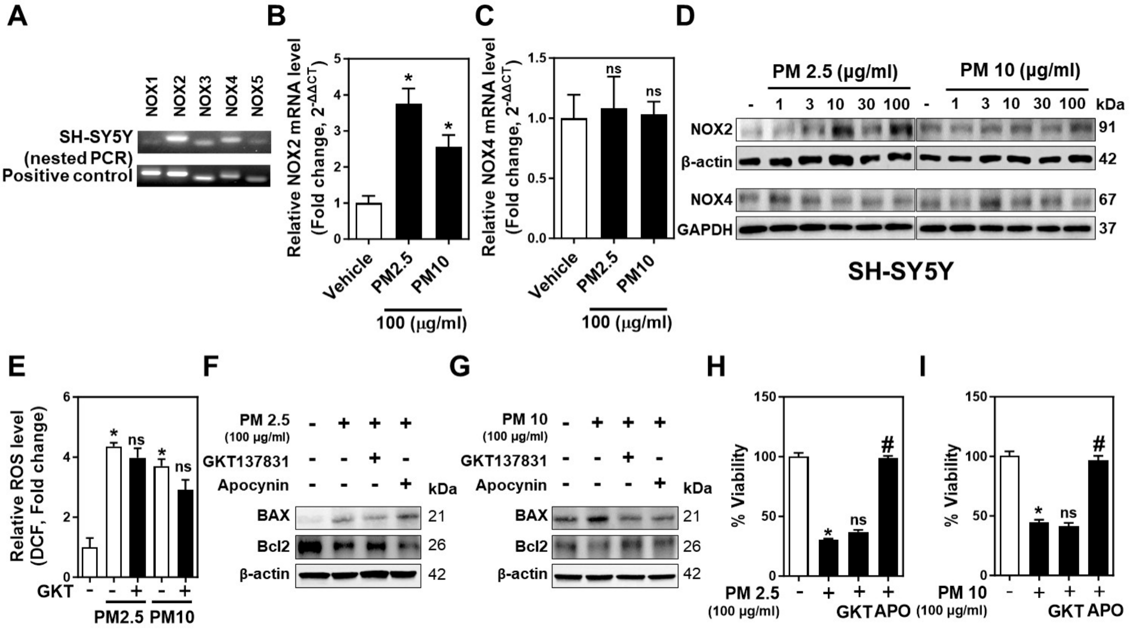

Supplementary Figure S3. PM2.5 and PM10 induce NOX2-mediated ROS production and apoptosis in SH-SY5Y. (A) Nested PCR following RT-PCR showing mRNA levels of human NOX1-5 in SH-SY5Y cells. cDNAs for the positive control were used A594 for NOX1-3, ACHN for NOX4, Ishikawa for NOX5. (B, C) Real-time qPCR for (B) NOX2 or (C) NOX4 mRNA in response to PM2.5 and PM10 (100 μg/ml) incubation for 24 h. PBS was used as vehicle control. (D) WB showing NOX2 and NOX4 expression after treatment with different doses of (upper) PM2.5 and (lower) PM10 (1, 3, 10, 30, 100 μg/ml) for 24 h. β-actin or GAPDH were used as a loading control. (E) GKT137831 (GKT, 10 μM for 10 min) suppressed PM2.5- and PM10-mediated (100 μg/ml for 1 h) cytosolic ROS (ΔDCF intensity) generation. (F, G) The protein expressions of BAX and Bcl2 were detected in SH-SY5Y cells, treated or not with GKT137831 (10 μM) and Apocynin (10 μM), in the presence or absence of (F) PM2.5 and (G) PM10 for 24 h. β-actin was used as a loading control. (H, I) Reduction of cell viability by (H) PM2.5 and (I) PM10 was recovered by treatment with GKT137831 (10 μM) and Apocynin (10 μM). The inhibitors were pretreated for 30 min before 24 h co-incubation with PM2.5 or PM10. All values are the mean ± SEM, *$p \le 0.01$ vs. Vehicle, #$p \le 0.01$ vs. PM2.5 or PM10, ns, non significant. One-way ANOVA was used for (B, C, E, H, and I).
