# Supplementary material for: Particulate Matter-Induced Neurotoxicity: Unveiling the Role of NOX4-Mediated ROS Production and Mitochondrial Dysfunction in Neuronal Apoptosis
Source: Int J Mol Sci. 2024 Jun 1;25(11):6116. doi: 10.3390/ijms25116116 (PMC11172693; doi:10.3390/ijms25116116)
Supplement: Supplementary file 1 [file ijms-25-06116-s001.zip › Supplementary Table S1.pdf]

Supplementary Table S1. List of primer sequences used for PCR

Gene (Accession no.)	Species	Primer sequence (5′ to 3′)		Size(bp)
18s (NR_145820.1)	M, H	Forward	AACCCGTTGAACCCCATT	149
		Reverse	CCATCCAATCGGTAGTAGCG	
NOX1 (NM_172203.2)	M	Forward	GTTTCTCTCCCGAAGGACCTCT	131
		Reverse	CCCCAACCAGGAAACCAGAAAC	
NOX2 (=Cybb) (NM_007807.5)	M	Forward	AAAGGTGGTCATCACCAAGG	88
		Reverse	TCCCACCTCCATCTTGAATC	
NOX3 (NM_198958.2)	M	Forward	TCTGAACGAGAGTGGGTCCT	138
		Reverse	GCCAATGCGGAACCCAGAA	
NOX4 (NM_015760.5)	M	Forward	CCACAGACCTGGATTTGGAT	91
		Reverse	TGGTGACAGGTTTGTTGCTC	
NOX1 (NM_007052.5)	H	Forward	GCGCCCTAAGTTTGAAGGG	114
		Reverse	ACAACCTTCTGCTGGGAGC	
NOX2 (=CYBB) (NM_000397.4)	H	Forward	AAGGCTTCAGGTCCACAGAGGAAA	103
		Reverse	AGACTTTGTATGGACGGCCCAACT	
NOX3 (NM_015718.3)	H	Forward	GATTGTTCGAGGCCAAACCC	142
		Reverse	AATCCATTTCCAAGCCGAGG	
NOX4 (NM_016931.5)	H	Forward	CCTCAACTGCAGCCTTATCC	84
		Reverse	CTCCTGCTTGGAACCTTCTG	
NOX5 (NM_024505.4)	H	Forward	CCGATAGAAGTGGCACCATCA	126
		Reverse	GCCGTGCACACACATCG	
